# Aerobic exercise promotes the functions of brown adipose tissue in obese mice via a mechanism involving COX2 in the VEGF signaling pathway

**DOI:** 10.1186/s12986-021-00581-0

**Published:** 2021-06-03

**Authors:** Pengyu Fu, Rongxin Zhu, Jie Jia, Yang Hu, Chengjun Wu, Pawel Cieszczyk, Hans-Christer Holmberg, Lijing Gong

**Affiliations:** 1 China Institute of Sport and Health Science, Beijing Sport University, Xinxi Road 48, Haidian District, Beijing, 100084 China; 2 Department of Physical Education, Northwestern Polytechnical University, West Youyi Road 127, Beilin District, Shaanxi, 710109 China; 3 Shanghai Research Institute of Sports Science, Xuhui District, Wuxing Road 87, Shanghai, 200030 China; 4 Sport Science College, Beijing Sport University, Xinxi Road 48, Haidian District, Beijing, 100084 China; 5 School of Biomedical Engineering, Dalian University of Technology and IC Technology Key Lab of Liaoning, Dalian, 116024 China; 6 Department of Molecular Biology, Faculty of Physical Education, Gdańsk University of Physical Education and Sport, ul. Kazimierza Górskiego 1, 80-336 Gdańsk, Poland; 7 Department of Physiology and Pharmacology, Biomedicum C5, Karolinska Institute, Stockholm, Sweden

**Keywords:** Aerobic exercise, Obesity, Brown adipose tissue, Gene expression profiling, VEGF signaling pathway, COX2

## Abstract

**Background:**

High-fat diet (HFD)-induced obesity causes immune cells to infiltrate adipose tissue, leading to chronic inflammation and metabolic syndrome. Brown adipose tissue (BAT) can dissipate the energy produced by lipid oxidation as heat, thereby counteracting obesity. Aerobic exercise activates BAT, but the specific underlying mechanism is still unclear.

**Methods:**

Male C57BL/6 J mice were divided into a normal diet control group (NC group) and HFD group (H group). After becoming obese, the animals in the H group were subdivided into a control group (HC group) and an exercise group (HE group, with treadmill training). After 4 weeks, the mRNA profile of BAT was determined, and then differentially expressed key genes and pathways were verified in vitro.

**Results:**

Relative to the NC group, the genes upregulated in the HC group coded mainly for proteins involved in immune system progression and inflammatory and immune responses, while the downregulated genes regulated lipid metabolism and oxidation–reduction. Relative to the HC group, the genes upregulated in the HE group coded for glycolipid metabolism, while those that were downregulated were involved in cell death and apoptosis. VEGF and other signaling pathways were enhanced by aerobic exercise. Interaction analysis revealed that the gene encoding cyclooxygenase 2 (COX2) of the VEGF signaling pathway is central to this process, which was verified by a sympathetic activator (isoprenaline hydrochloride) and COX2 inhibitor (NS-398).

**Conclusions:**

In mice with HFD-induced obesity, four weeks of aerobic exercise elevated BAT mass and increased the expression of genes related to glycolipid metabolism and anti-inflammatory processes. Several pathways are involved, with COX2 in the VEGF signaling pathway playing a key role.

## Background

Obesity characterized by increased adipocyte size and excessive accumulation of fat, has become one of the major risk factors for metabolic syndrome (MS) [1].

Obesity induced by a high-fat diet (HFD), the major environmental cause of this condition [2], leads to immune cell infiltration into white adipose tissue (WAT) and the release of proinflammatory factors to other tissues, which can result in chronic inflammation. In contrast to energy storage by WAT, brown adipose tissue (BAT) converts chemical energy to heat through uncoupling of mitochondrial oxidative phosphorylation to maintain body temperature [2–5].

BAT is characterized by abundant expression of uncoupling protein 1 (UCP1), which uncouples mitochondrial respiration from adenosine triphosphate (ATP) synthesis, increasing the proton leakage across the inner mitochondrial membrane and releasing the proton motive force as heat to rapidly activate thermogenesis rather than driving ATP synthase. Obesity might influence BAT, including reducing its thermogenic activity and capacity to utilize free fatty acids (FFAs) [6]. However, it is unclear whether the chronic inflammatory state associated with obesity is related to the decrease in the mass and function of BAT.

Numerous investigations have demonstrated that exposure to cold stimulates BAT effectively. The primary underlying mechanism involves activation of the sympathetic nervous system (SNS) to release norepinephrine and activate the β-adrenergic receptor (β-AR) [7]. Moreover, exercise, which effectively reduces fat and helps prevent obesity [8], also activates the SNS [9] and has been shown to stimulate BAT.

In contrast, some recent studies have concluded that exercise attenuates the activity of BAT [10], proposing instead that exercise itself is thermogenic and thereby unlikely to enhance thermogenesis by BAT [11]. In agreement with this interesting hypothesis, some researchers have reported that aerobic exercise does not alter the expression of UCP1 in this tissue [12]. However, it is gradually becoming apparent that BAT has other functions as well, some of which might be influenced by exercise.

In light of the general belief that adaptations in BAT contribute to the beneficial effects of exercise on metabolic health, it is of considerable importance to explore all potential responses of this tissue to aerobic exercise [13]. In this context, profiling gene expression should provide invaluable insights. Accordingly, we employed mRNA microarrays to profile the genes expressed by the BAT of normal mice and obese mice with and without aerobic exercise.

## Methods

### Animal model experiment

**Fig. 1:**
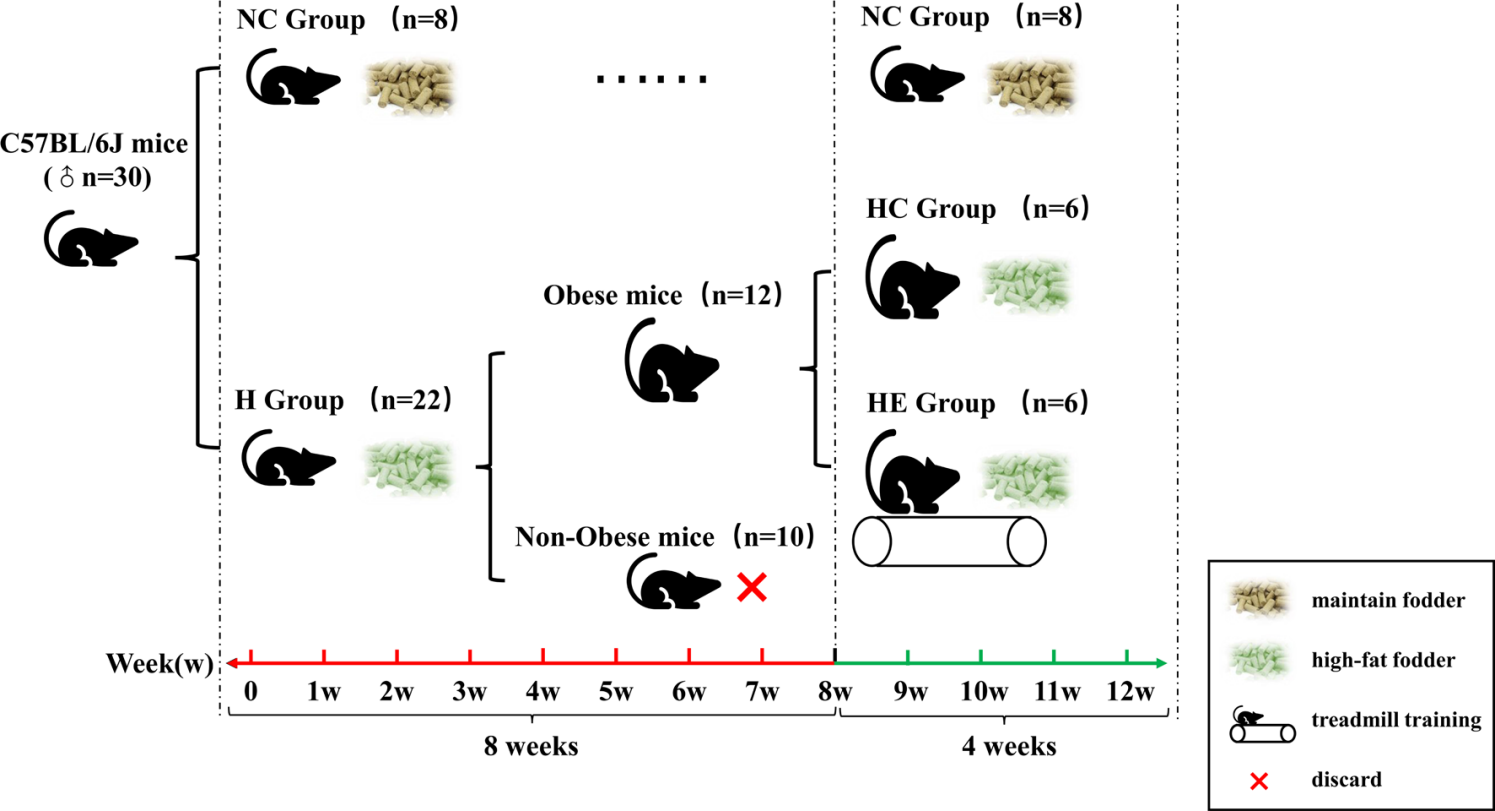
Illustration of the grouping and intervention procedures

Thirty 4-week-old male C57BL/6 J mice (Beijing Vital River Experimental Animal Technology Co. Ltd, China) weighing 8.2 ± 0.1 g each were housed singly with a 12-h light/12-h dark cycle at 22 ± 2℃ and 50–70% humidity. These animals were divided into a normal diet control group (3.82 kcal/g; 11.85% of energy from fat, 23.07% from protein and 65.08% from carbohydrate; Beijing Keao Xieli Feed Co., Ltd.) (NC group, n = 8) and a HFD group (H group, n = 22), fed high-fat chow (4.46 kcal/g; 40% of energy from fat, 20% from protein and 40% from carbohydrate; D12109C, Research Diet Inc., USA) for 8 weeks. Obesity was considered to have been established in the H group when the animals weighed 20% more than the average weight of the NC group, a criterion based on human studies and commonly applied to mice [14, 15]. The nonobese mice (n = 10) were sacrificed under anesthesia by bleeding from the heart, and the obese mice (n = 12) were subdivided into the obese control (HC group, n = 6) and obese exercise groups (HE group, n = 6). The training program of the HE group involved running on a flat treadmill at a speed of 10 m/min, 1 h/day, 6 times each week for 4 weeks, during which both the HC and HE groups continued to receive high-fat chow. The grouping and intervention are illustrated in Fig. 1. The body weight and food intake of each animal were recorded every week. All animal experiments were preapproved by the Beijing Sports University Science Ethics Committee (No. 2015040) and carried out in strict accordance with the Guide for the Care and Use of Laboratory Animals (Washington, USA).

### Body composition and BAT mass

Body composition was determined with an animal composition analyzer (EchoMRI™-700, U.S.A.). After intraperitoneal injection of 2% pentobarbital sodium, BAT mass was measured by a small-animal microscopic CT (microCT-LCT200, HITACHI ALOKA, Japan), and the BAT ratio (BAT/total fat content × 100%, B/F%) was calculated.

### Slaughter

The mice were fasted for 12 h and then anesthetized with 2% pentobarbital sodium. The animals were then sacrificed by collecting cardiac blood, and the serum was frozen at -20℃. BAT collected from the scapula was divided into two parts, one placed in RNA extraction medium and the other stored at -80℃.

### Biochemical indicators in serum

The serum levels of triglyceride (TG), total cholesterol (TC) and glucose (GLU) were measured by an Automatic Biochemical Analyzer (7020, HITACHI Limited, Japan).

### RNA extraction and fluorescent labeling

Total RNA of BAT was extracted using the Ambion mirVana miRNA Isolation Kit (USA) and purified using Ambion NucleoSpin® RNA clean-up (MACHEREY–NAGEL, Germany), after which the RNA was quantified by spectrophotometry, and its quality was analyzed by denaturing formaldehyde gel electrophoresis. The purity (A260/280 ≥ 1.90), quantity (≥ 10 µg) and integrity of this total RNA were adequate for gene expression profiling. Total cDNAs prepared from the total RNA using random primers were labeled during reverse transcription, purified with the PCR Nucleo Spin Extract II Kit (MN), hybridized at 45℃ overnight, washed with a mixture of 0.2% SDS and 2 × SSC for 5 min at 42℃ and thereafter with 0.2 × SSC for 5 min at room temperature, and the microarray slides were finally dried and scanned.

### Microarrays and data analyses

Three mice randomly selected from each group were used for the microarrays. BAT gene expression was analyzed using the Mouse (V2) Gene Expression Microarray (8 × 60 K mRNA single-channel microarray) with scanning by the Agilent G2565CA Microarray Scanner. Microarray images were converted into digital signals with Feature Extraction image analysis software, and the original data were put into GeneSpring GX software to normalize the signal by percentile shift. Thereafter, genes demonstrating an absolute fold-change (abs) ≥ 2 were marked as differentially expressed. Cluster analysis and graphical display were achieved with Cluster 3.0 software. Gene ontology (GO) analysis and pathway analysis of differentially expressed genes were performed utilizing KOBAS 2.0 (KEGG Orthology Based Annotation System) and DAVID Bioinformatics Resources 6.7. Employing the GO (http://www.geneongoloty.org/) database, these genes were classified according to the biological process (BP, from the beginning to the end of a molecular event, including the function of cells, tissues, organs, and species) in which they were involved. Analysis of gene-to-gene interactions was performed by String and Cytoscape.

### Reverse transcription-quantitative PCR (RT-qPCR) to determine relative mRNA expression

Total RNA was extracted from BAT using the TaKaRa MiniBEST Universal RNA Extraction Kit and reverse transcribed into cDNA with PrimeScript RT Master Mix (TaKaRa, Japan). SYBR Premix Ex TaqII (TaKaRa, Japan) was utilized to run the RT-qPCR assay in the ABI 7500 Real-Time PCR system. Melt curve analysis was applied to determine whether any unwanted reaction product was present. 18S ribosomal RNA was the internal reference. Quantification was achieved by the 2-ΔΔCt method. Primer sequences were designed using Oligo 7 software, and primer specificity was assured with the National Center for Biotechnology Information (NCBI)’s online primer design tool (Table 1).

**Table 1:** The sequences of the primers employed for RT-qPCR

Gene Symbol	Forward primer sequence (5′–3′)	Reverse primer sequence (3′–5′)
Acsl5	GCCTGAAATCCTTTGAGCAG	GGCAAGCTCTACTCGTTTGG
Fasn	TGCTCATCCACTCAGGTTCA	AGGTATGCTCGCTTCTCTGC
Fabp5	ACGACTGTGTTCTCTTGTAACCTG	TCTCCTTCCCGTCCCATT
Acaca	TCTCCTTCCCGTCCCATT	TTGTTTGGTCGTGACTGCTC
Fbp1	ACATCGTTCCCACCGAGAT	CACTTGGCTTTGTGCTTCCT
Ptges	GATGAGGCTGCGGAAGAA	CCGAGGAAGAGGAAAGGATAG
Ptgis	GCCTTCTCCTCTTTCCCTTC	GCCGTTTCCCATCTTTGTAA

### Brown adipocyte culture and adipogenesis

C_3_H_10_T_1/2_ and clone 8 cells (kindly provided by Cell Bank, Chinese Academy of Sciences, Shanghai, China) [16, 17] were maintained in DMEM/F12 medium with 10% fetal bovine serum (Gibco, USA) in a humidified incubator at 37 °C and under 5% CO_2_. Brown adipogenesis was achieved as described previously. In brief, the cells were first cultured in differentiation medium [DMEM/F12 containing 10% fetal bovine serum, 20 nM insulin and 1 nM 3,3′5-triiodo-L-thyronine (T3)] for 4 days, with the medium being changed every other day. On day 4, confluent cells were treated with a brown adipose adipogenic cocktail (differentiation medium containing 2 μg/mL dexamethasone, 0.5 mM isobutyl methylxanthine, 0.125 mM indomethacin and 1 μM rosiglitazone) for 2 days, after which this medium was replaced by differentiation medium and changed every other day. On day 10, the fully differentiated adipocytes were treated with 10 μM isoprenaline hydrochloride [for activation of β-adrenaline 3 (Adrβ3)] and/or 100 μM NS-398 [a selective cyclooxygenase 2 (COX2) inhibitor] for 6 h. The brown adipocytes thus obtained were divided into four groups: the control group (C group), the isoprenaline intervention group (ISO group), the NS-398 intervention group (NS group) and the intervention group receiving isoprenaline and NS-398 (NS-ISO group).

### Observation of lipid droplets by oil red O staining

At the end of the drug treatment, the cells were fixed with 4% paraformaldehyde for 5 min, after which oil red O dye solution (500 μL per well in 24-well plates) was added for 15 min; rinsing and decolorizing were performed with 60% isopropyl alcohol; and rinsing with distilled water was carried out 3 times. Finally, 500 μL of PBS was added as a sealant, and the cells were photographed under an inverted microscope (DMI4000 B, LEICA, Germany).

#### Measurement of glycerol

After 6 h of intervention, the level of glycerol in the cell culture medium was tested in accordance with the instructions provided with the kit (10,011,725, Cayman). A Bio-Rad xMark microporous plate spectrophotometer was used to determine the absorbance of each well at 562 nm, and the glycerol content was calculated.

#### Western blotting

Protein was extracted from BAT by homogenizing with RIPA buffer. Protein was extracted from brown adipocytes using the Minute Total Protein Extraction Kit (Invent, USA). In each lane, 20 μg (for tissue samples) or 10 μg of protein (for cell samples) was initially separated on a Bolt 4–12% Bis–Tris plus gel (Invitrogen, USA) and then transferred to nitrocellulose filter membranes with the iBlot 2 Gel Transfer Device (Life Technologies, USA) using iBlot 2 NC Regular Stack (Invitrogen, USA). After 30 min of blocking in 5% bovine serum albumin (BSA), the membranes were incubated with the appropriate primary and corresponding secondary antibodies: rabbit anti-Adrβ3 (ab8412, Abcam), -VEGFa (ab46154, Abcam), -Flk1 (ab1193, Abcam), -PLCγ (ab76155, Abcam), -COX2 (12375-1-AP, Protein Tech), -UCP1 (ab23841, Abcam), and -PGC-1α (ab54481, Abcam) and mouse anti-β-actin (A5441, Sigma), followed by visualization with SuperSignal West Femto Maximum Sensitivity Substrate (Thermo Scientific, USA) and capture with the ChemiDoc XRS + System (Bio-Rad, USA). The intensity of each band was quantified using Image Lab software (Bio-Rad, USA) and normalized against β-actin.

#### Statistical analysis

Statistical analysis was performed with SPSS 19.0 and GraphPad Prism 8 software, and the values presented are the mean ± SEM. The independent sample t-test was applied to compare the indices between the two groups, while multigroup indices were compared by two-way analysis of variance (ANOVA). *P* < 0.05 or < 0.01 was considered statistically significant.

## Results

### Food intake, degree of obesity, BAT mass and UCP1 expression

**Fig. 2:**
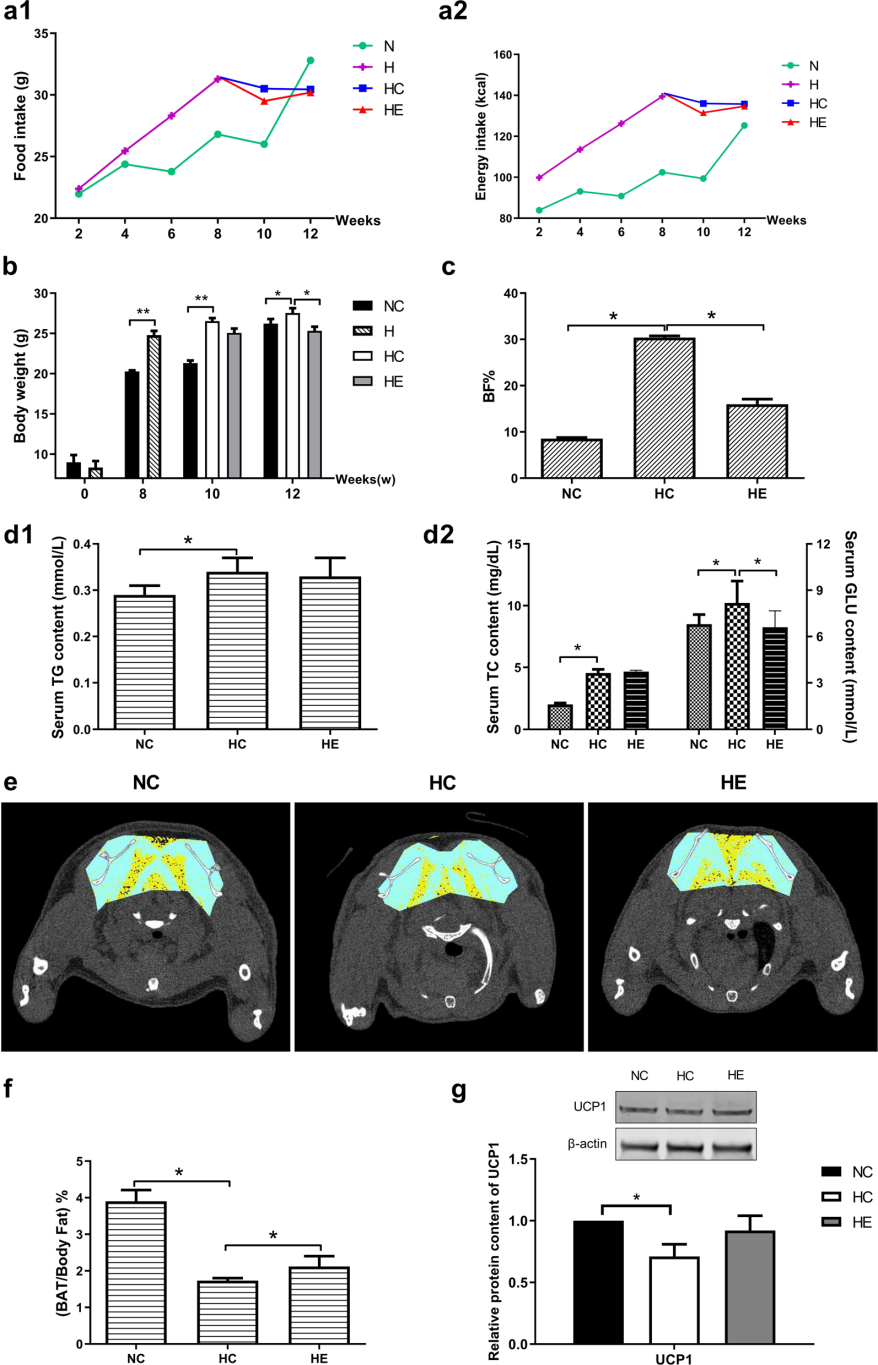
Food intake, degree of obesity, BAT mass and UCP1 expression in the different groups of mice. Food intake (a1), energy intake (a2) and body weight (**b**) during feeding and intervention (0–12 weeks); Body fat percentage (BF%) after the 4-week intervention; Serum TG (d1), TC and GLU (d2) levels in each group of mice after intervention; **e** CT scans where yellow is WAT, blue fascia and muscle, white bone, and black BAT (indicated by the black arrows); **f** BAT mass percentage [(BAT mass/BF) %] after the 4-week intervention; **g** western blotting of UCP1 and the relative protein content in BAT after the intervention. *P < 0.05 and **P < 0.01 represent statistical significance. n = 6

Prior to the intervention, the mice in all groups had similar body weights (8.2 ± 0.1 g). After 8 weeks on an HFD, obesity was established. The lack of any significant difference in food intake and energy intake (single-week total) by the groups during training indicated that the biological effect of exercise was not caused by the inhibition of food intake and energy intake (Fig. 2a1, a2). After the 4-week intervention, the HC group weighed more and had a higher body fat percentage (BF%) than the NC and HE groups (Fig. 2b, c). After 12 weeks of feeding, the serum levels of TG, TC and GLU in the HC group were higher than those in the NC group. After 4 weeks of exercise, the GLU level in the HE group was lower than that in the HC group (*P* < 0.05) (Fig. 2d1, d2). CT scanning revealed the mass and location of BAT (Fig. 2e). After the 4-week intervention, the HC group had less BAT mass percentage [(BAT mass/BF) %] than the NC group, while the HE group had less than the NC group but significantly more than the HC group (*P* < 0.05) (Fig. 2f). After the 4-week intervention, the protein expression of UCP1 in the BAT of the HC group was lower than the NC group (*P* < 0.05), and the HE group tended to have more UCP1 than the HC group, although this difference was not statistically significant (Fig. 2g).

### Microarray data analysis and validation

**Fig. 3:**
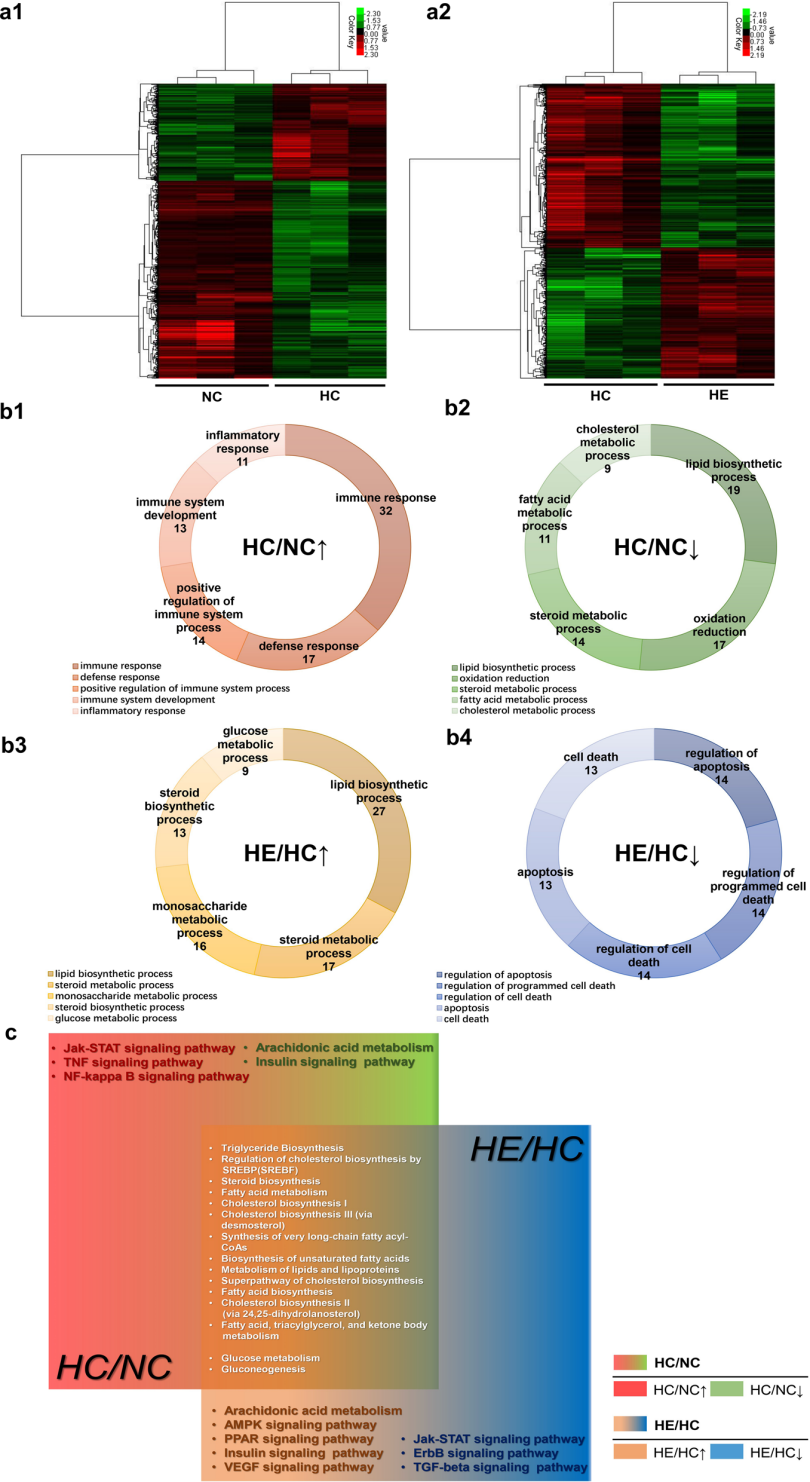

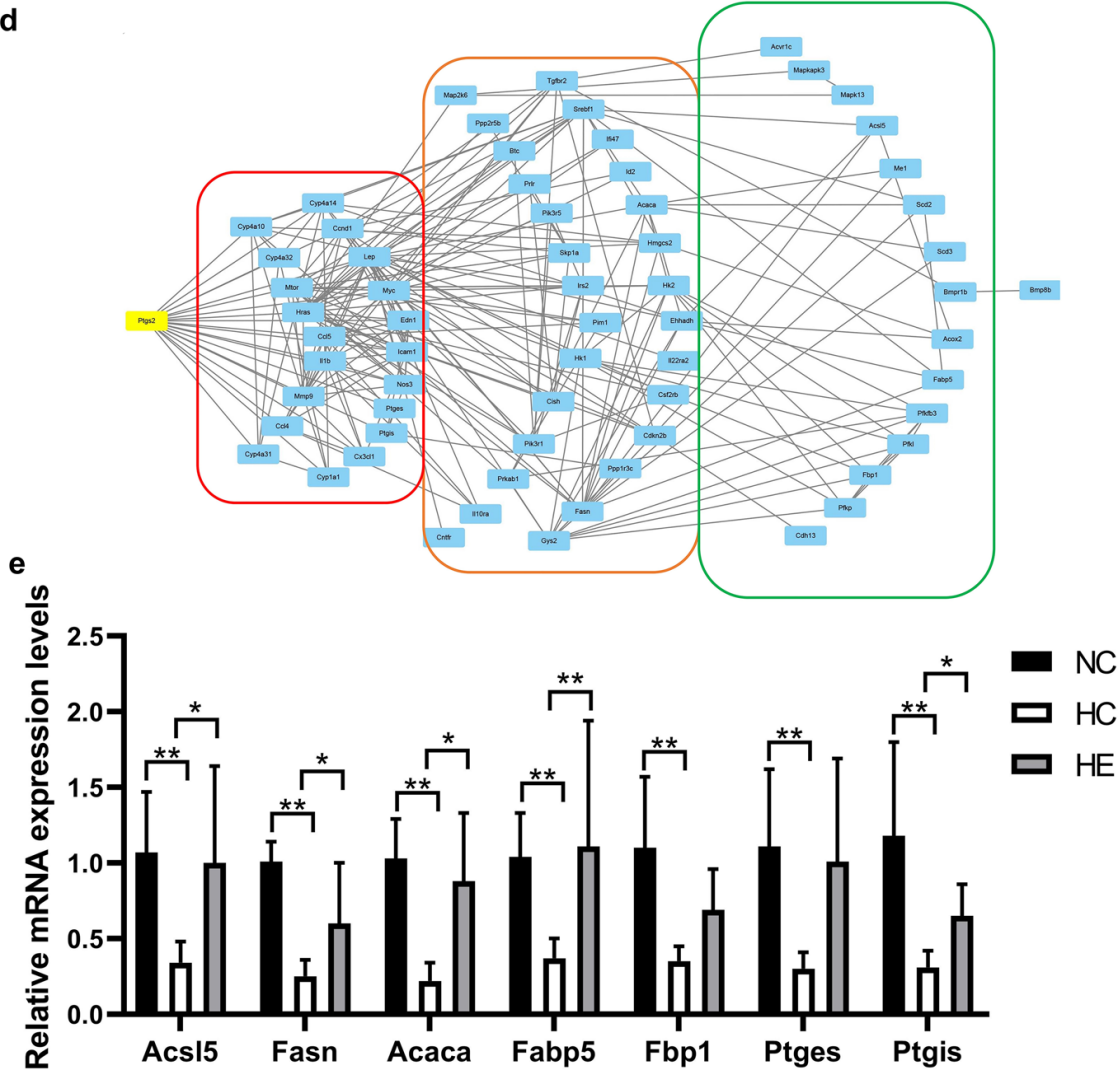
Microarray analysis of BAT in the different groups of mice and validation. Clusters of the differentially expressed genes in the HC versus NC groups (a1) and the HE versus HC groups (a2). Green represents downregulation relative to the mean level of the control group, and red represents upregulation. Black indicates missing or excluded data. GO analysis of the differentially expressed genes (b1, b2, b3 and b4). Comparison of the top biological process (BP) associated with the differentially expressed genes in the HC versus NC (HC/NC) and HE versus HC (HE/HC) groups. The figures show the BP enriched in the top five differential genes (ranked by input gene number) in each group. HC/NC↑/↓ indicates that the HC group was higher/lower than the NC group, and HE/HC↑/↓ indicates that the HE group was higher/lower than the HC group, both here and in the following. **c** Wayne diagram of the major pathways enriched in the differentially expressed genes. Red (region and text) represents genes that are upregulated in the HC/NC group, and green represents genes that are downregulated. Orange represents genes that are upregulated in both HC/NC and HE/HC groups downregulated. The white text depicts genes whose levels of expression did not differ. **d** The interaction diagram and the COX2 control network diagram of the differentially expressed genes. The differentially expressed genes in the red frame have a primary regulatory relationship with Ptgs2/COX2; the orange frame contains the secondary regulatory genes, while the green frame contains the tertiary regulatory genes. **e** RT-qPCR verification of key differentially expressed genes. *P < 0.05 and **P < 0.01 represent statistical significance. n = 3

The results of clustering analysis are depicted in Fig. 3a1, a2. Gene expression was compared for the HC versus NC and HE versus HC groups. Relative to the NC group, 386 genes were upregulated and 467 were downregulated in the HC group, and relative to the HC group, 486 genes were upregulated and 286 were downregulated in the HE group. The genes upregulated in the HC group were involved primarily in immune system progression and inflammatory and immune responses (Fig. 3b1), while the downregulated genes were associated mainly with lipid metabolism and oxidation reduction (Fig. 3b2). The genes upregulated in the HE group were involved primarily in glycolipid metabolism (Fig. 3b3), while the downregulated were associated mainly with cell apoptosis and death (Fig. 3b4) [Gene Ontology (GO) enrichment analysis].

Pathway analysis revealed that most of the genes upregulated in the HE group and downregulated in the HC group were related to glycolipid metabolism. In the case of HC, the Jak-STAT, TNF and NF-kappa B signaling pathways were upregulated. The pathways downregulated in HC included arachidonic acid metabolism and insulin signaling. Pathways upregulated in HE were associated with arachidonic acid metabolism and the AMPK, PPAR, insulin and VEGF signaling pathways, while those downregulated included the Jak-STAT, ErbB and TGF-beta signaling pathways (Fig. 3c). The differentially expressed genes and their pathways are shown in Table 2 [Kyoto Encyclopedia of Genes and Genomes (KEGG) Pathway database (http://www.genome.jp/kegg/) and three additional databases (Reactome, BioCyc and Panther)].

**Table 2:** Pathway analysis of the differentially expressed genes (*P* < 0.05)

Comparison	Major Enriched Pathways	Associated Genes
HC versus NC↑	Jak-STAT signaling pathway	Prlr, Pik3r5, Pim1, Cntfr, Csf2rb, Il22ra2, Il10ra
	TNF signaling pathway	Cx3cl1, Icam1, Pik3r5, Map2k6, Ifi47, Edn1, Il1b, Ccl5, Mapk13, Mmp9
	NF-kappa B signaling pathway	Il1b, Lbp, Icam1, Bcl2a1d, Ccl4
HC versus NC↓	Arachidonic acid metabolism	Ptgr1, Ptgis, Cyp4a14, Ptges
	Insulin signaling pathway	Fasn, Acaca, Pik3r5, Mtor, Fbp1, Ppp1r3c, Srebf1, Hras
HE versus HC↑	Arachidonic acid metabolism	Cyp1a1, Cyp4a32, Ptges, Cyp4a31, Cyp4a10, Ptgr1, Ptgis, Cyp4a14, Ptgs2
	AMPK signaling pathway	Lep, Pfkfb3, Ccnd1, Pfkl, Pik3r5, Pfkp, Scd2, Prkab1, Fasn, Fbp1, Scd3, Gys2, Acaca, Ppp2r5b, Pik3r1, Irs2
	PPAR signaling pathway	Me1, Scd3, Ehhadh, Hmgcs2, Cyp4a32, Cyp4a31, Fabp5, Cyp4a10, Acsl5, Cyp4a14, Acox2, Scd2
	Insulin signaling pathway	Pik3r1, Fasn, Acaca, Pde3a, Prkab1, Fbp1, Pik3r5, Gys2, Hk1, Hk2, Cblc, Irs2
	VEGF signaling pathway	Pik3r5, Pik3r1, Nos3, Mapkapk3, Ptgis, Ptgs2
HE versus HC↓	Jak-STAT signaling pathway	Lep, Myc, Ccnd1, Pik3r5, Pik3r1, Cish, Cntfr, Il22ra2, Cblc
	ErbB signaling pathway	Zfyve28, Cdh13, Cblc, Btc
	TGF-beta signaling pathway	Myc, Cdkn2b, Id2, Acvr1c, Skp1a, Bmp8b, Bmpr1b, Tgfbr2

Janus kinases/signal transducer and activator of transcription (JAK/STAT); Tumor necrosis factor (TNF); Nuclear factor-kappa B (NF-kappa B); Adenosine 5’-monophosphate (AMP)-activated protein kinase (AMPK); Receptor tyrosine-protein kinase erbB isoform b (ErbB); Peroxisome proliferator-activated receptor (PPAR); Vascular endothelial growth factor (VEGF); Transforming growth factor-beta (TGF-beta). n = 3

Analysis by String and Cytoscape revealed that prostaglandin-endoperoxide synthase 2/cyclooxygenase 2 (Ptgs2/COX2) in the VEGF signaling pathway plays a central role in the regulation of the differentially expressed genes, which are also closely related to glycolipid metabolism, thermogenesis and inflammation of BAT (Fig. 3d). To confirm these findings, the expression of acyl-CoA synthetase long-chain family member 5 (Acsl5), fatty acid synthase (Fasn), acetyl-coenzyme A carboxylase alpha (Acaca), fatty acid binding protein 5 (Fabp5), fructose bisphosphatase 1 (Fbp1), prostaglandin E synthase (Ptges) and prostaglandin I2 (prostacyclin) synthase (Ptgis) was tested by RT-qPCR. The relative expression of all seven of these genes was lower in the HC group than in the NC group (*P* < 0.01), and the expression of Acsl5, Fasn, Acaca, Fabp5 and Ptgis in the HE group was significantly higher than that in the HC group (*P* < 0.05) (Fig. 3e).

### The VEGF signaling pathway and verification of related protein expression

**Fig. 4:**
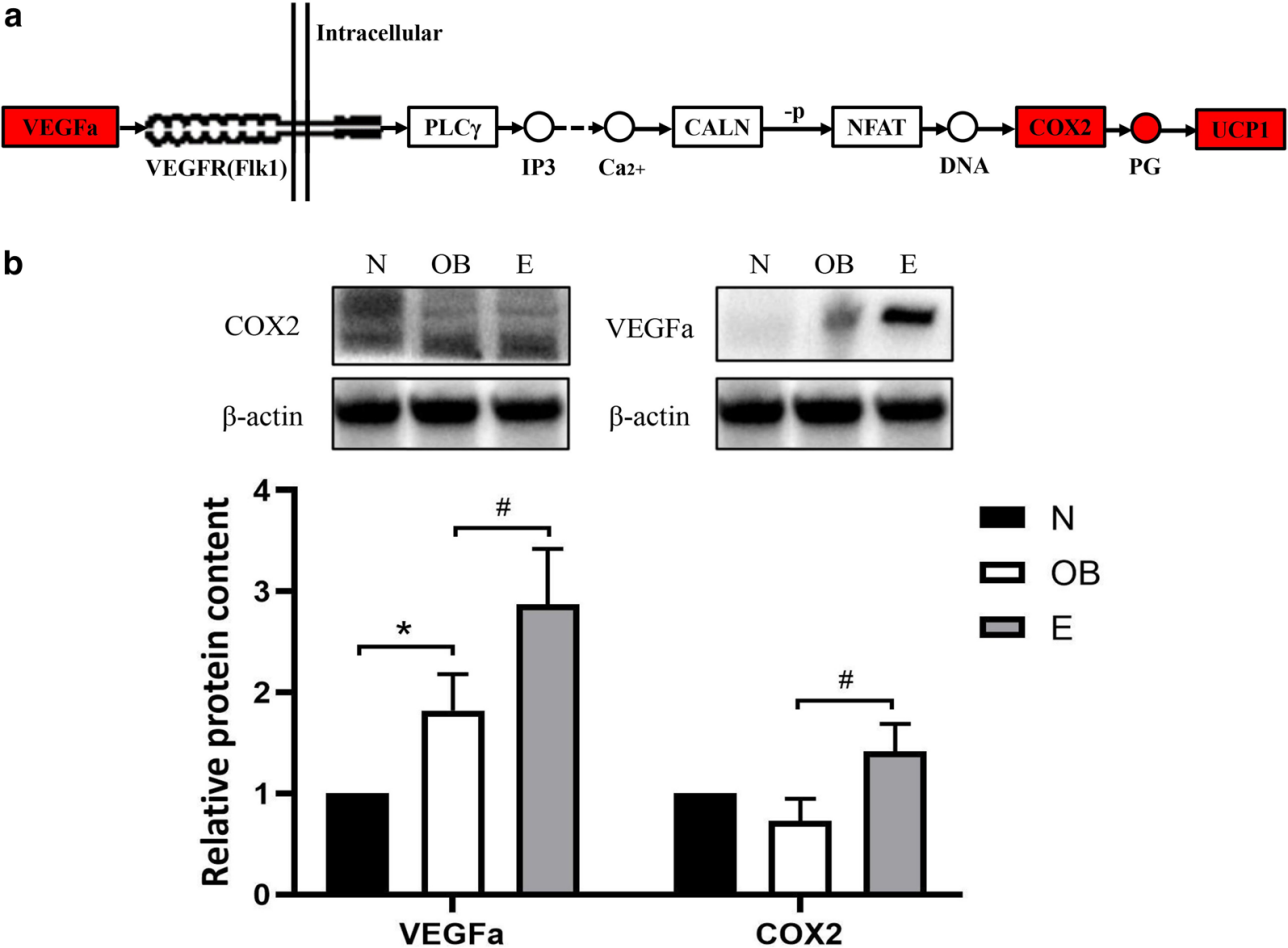
The VEGF signaling pathway in the BAT of the different groups of mice and verification. **a** VEGF signaling pathway and KEGG analysis of this pathway and related genes. Red represents the upregulated genes. Solid arrows indicate a direct action, dashed arrows indicate indirect action; “ − *P*” represents dephosphorylation. **b** Levels of the VEGF and COX2 proteins. *P < 0.05 and **P < 0.01 represent statistical significance. n = 3

On the basis of KEGG analysis, we propose that the following cascade events occur in the VEGF signaling pathway: VEGFa binds to one of its receptor forms related to tyrosine kinase 1 (Flk-1); activates phospholipase C gamma (PLCγ) to release inositol trisphosphate (IP3) and Ca^2+^; increases the expression of protein phosphatase 3, catalytic subunit, alpha isoform (CALN), which dephosphorylates the activated nuclear factor of activated T cells (NFAT); and then enhances the expression of COX2 and prostaglandin (PG) to activate UCP1 and promote thermogenesis by BAT (Fig. 4a). The levels of VEGFa and COX2 proteins in the VEGF signaling pathway were tested by western blotting, and the level of VEGFa was found to be higher in the HC group than in the NC group, while the levels of VEGF and COX2 in the HE group were significantly higher than those in the HC group (*P* < 0.05) (Fig. 4b).

### Functional verification of the role of COX2 in brown adipocytes

**Fig. 5:**
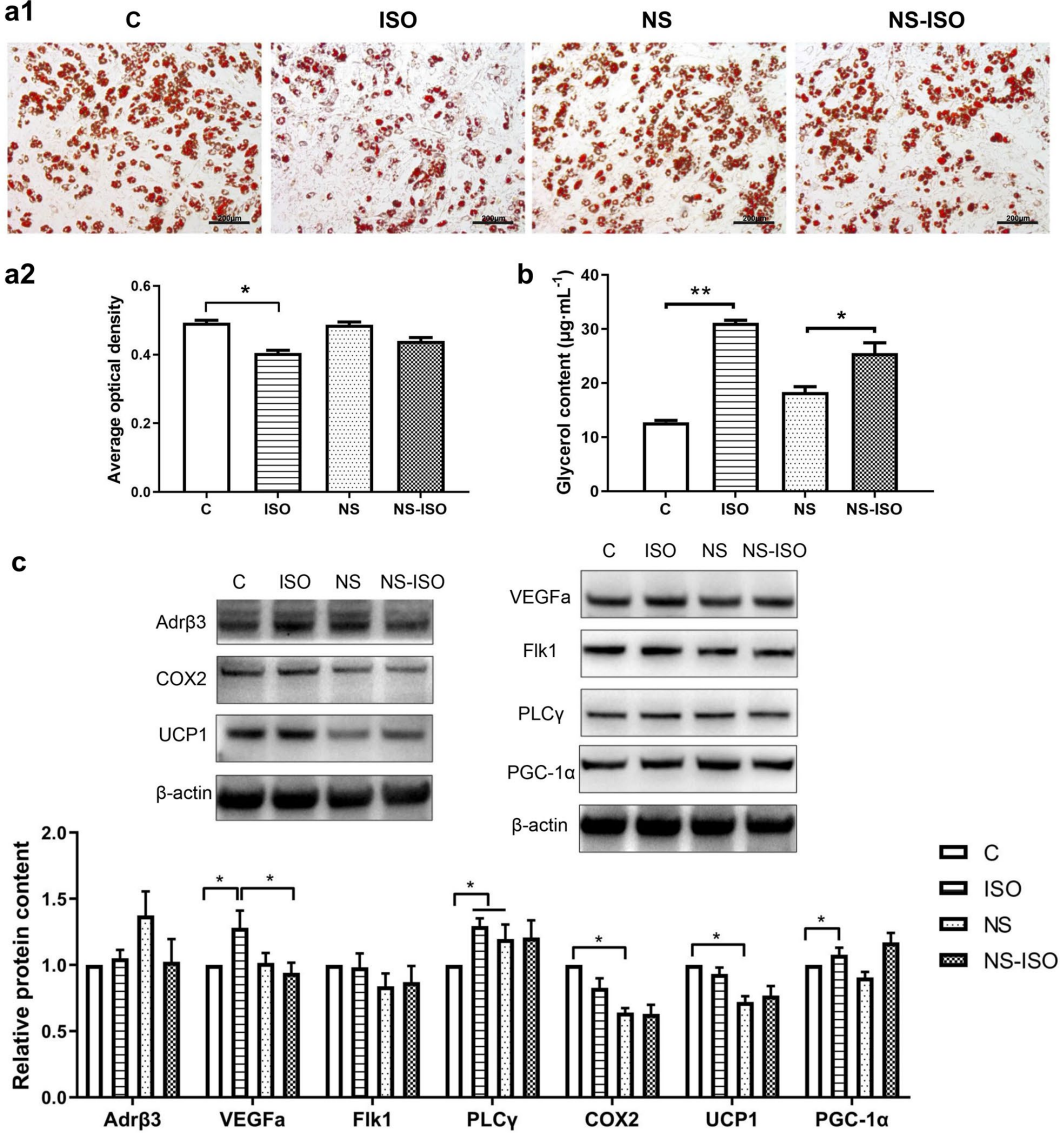
Functional verification of the role of COX2 in brown adipocytes. (a1) Oil red O staining of lipid droplets in brown adipocytes (scale bar = 200 μm) and (a2) the AOD levels of each group. **b** The glycerol contents of brown adipocytes after treatment with ISO and/or NS. **c** Effects of these compounds on the levels of COX2 and other components of the VEGF signaling pathway (Adrβ, VEGFa, Flk1, PLCγ, COX2, UCP1 and PGC-1α) in brown adipocytes. *P < 0.05 and **P < 0.01 represent statistical significance

Oil red O stains the lipid droplets in adipocytes, the main location of triglycerides. After the brown adipocytes were treated with isoprenaline for 6 h, staining with oil red O became lighter, and the size and quantity of lipid droplets and average optical density (AOD) both decreased. There was no significant difference with or without NS-398 treatment. After treatment with NS-398 and isoprenaline for 6 h, staining with oil red O and the size of intracellular lipid droplets were lower than those in the NS group, but the extent of decrease was less than that in the ISO group (*P* < 0.05) (Fig. 5a1, a2).

TGs are hydrolyzed by lipase to produce fatty acids (FAs) and glycerol, the latter being released into the blood and reflecting the extent of lipid decomposition. Isoprenaline increased the content of glycerol in the media, while NS-398 had no effect. The median glycerol content in the NS-ISO group was higher than that in the NS group (Fig. 5b).

The levels of VEGFa and peroxisome proliferator-activated receptor γ coactivator-1α (PGC-1α) in the ISO group were higher than those in the C group, while the PLCγ levels in the ISO and NS groups were significantly higher than those in the C group (*P* < 0.05). The levels of COX2 and UCP1 in the NS group were significantly lower than those in the C group (*P* < 0.05). The levels of VEGFa and PGC-1α in the NS-ISO group were significantly lower than those in the ISO group (*P* < 0.05) (Fig. 5c).

## Discussion

### The relationship between obesity and BAT

Here, 8 weeks of exposure to a HFD caused mice to become obese, and continued feeding with this same diet for 4 more weeks increased their BF% and serum levels of TG, TC and GLU. Since active BAT was found in adult humans, a growing number of studies confirmed that activation of BAT has an anti-obesity effect, and consequently, functional changes in the BAT of obese organisms have attracted considerable attention [16].

Our study found that obese mice have a significantly reduced BAT percentage and UCP1 expression, and microarray analyses showed that obesity was associated with reduced glycolipid metabolism. This observation is consistent with the findings by Kim and colleagues that the expression of genes related to sterol biosynthesis and FA metabolism in the BAT of mice decreased continuously with the period of exposure to a HFD (maximal at 24 weeks) [17]. BAT oxidizes excess fat and glucose for thermogenesis. After FAs enter the mitochondria in brown adipocytes, they can be utilized to produce heat through UCP1 or stored in lipid droplets by reesterification to TG [18, 19]. BAT plays an important role in the catabolism of TG and is considered to be among the organs primarily responsible for clearing lipids from the plasma [20]. Activation of UCP1 and fatty acid β-oxidation play a key role in this context [21]. Thermogenesis by 63 g of BAT in the clavicle of a healthy adult could be equivalent to a 4.1-kg weight loss [3]. The body weight, fat mass, Lee’s index and TG levels in the serum of mice in which BAT had been deleted were increased significantly after 16 weeks on a HFD [22]. The fasting blood glucose levels of Chinese adults with active BAT (aBAT) were significantly lower than those of other subjects [23]. All available evidence indicates that a decrease in BAT mass or activity can lead to increases in serum TC and glucose levels [24].

Our microarray results also revealed that obesity promotes immune system progression and inflammatory and immune responses in BAT, which is consistent with the observations by Kim and Svahn and coworkers [4, 17]. Obesity can cause immune cells to infiltrate adipose tissue and release proinflammatory factors such as cytokines to other tissues, which aggravates metabolic disorders and results in chronic inflammation [25]. The infiltration of activated macrophages into tissues is the key inflammatory event. BAT can inhibit inflammatory macrophages, while WAT enhances the inflammatory response [26]. On the other hand, the elevated levels of inflammatory factors associated with obesity inhibit the activation and recruitment of BAT, as well as the release of inflammatory factors from this tissue. In addition, activation of the complement system stimulates the secretion of leptin, which in turn causes proinflammatory factors to accumulate at inflammatory sites in a vicious cycle [27]. This process is also associated with the production of free radicals, which decrease the expression of BAT-specific genes and the activity of BAT [28].

### Aerobic exercise activates BAT in obese mice

The enhanced excitability of the SNS could stimulate BAT activity, inducing and/or inhibiting specific genes that regulate the differentiation and proliferation of brown adipocytes [12]. Thus, aerobic exercise, which effectively elevates SNS activity, was once thought to activate BAT, and regular exercise might promote the recruitment and relative number of brown adipose precursor cells [29]. In connection with previous investigations, the level of expression of thermogenesis-related genes such as UCP1 was used as an indicator of the degree of BAT activation, but recent studies have found that activation of BAT by aerobic exercise may not depend on UCP1 expression [30]. We found that even with a continued HFD, aerobic exercise reduced body weight, body fat percentage and blood glucose levels and elevated the BAT percentage in obese mice. Although aerobic exercise failed to enhance the expression of UCP1 in BAT, glycolipid metabolism was increased, and genes associated with cell apoptosis and death were downregulated.

Moreover, aerobic exercise upregulated the expression of genes involved in pathways related to glucose metabolism (insulin/AMPK signaling pathway) and lipid metabolism (PPAR/VEGF signaling pathway and arachidonic acid metabolism) in BAT and downregulated pathways related to inflammatory responses (the Jak-STAT/ErbB/TGF-beta signaling pathway). These observations indicate that exercise not only activates glycolipid metabolism by BAT but also may influence the effects of obesity on chronic inflammation. Running slightly promotes thermogenesis by BAT (with no difference in the level of UCP1) while influencing the morphology, sympathetic tone and vascularization of this tissue [30]. Enhancement of insulin signaling, together with inhibition of JNK activation and of the production of proinflammatory cytokines by BAT, may be involved in the ability of exercise to alleviate obesity [31]. Exercise could also counteract obesity through adrenergic recruitment of brown adipocytes, with their endocrine support of angiogenesis [30]. In the future, the production of WAT browning or formation of beige/brite cells in response to aerobic exercise by obese organisms should be examined to further clarify the mechanism by which exercise regulates BAT.

### Exercise upregulates the VEGF-COX2 pathway in BAT in obese mice

By analyzing the interaction of differentially expressed genes in the key regulatory pathways, we found that COX2 in the VEGF signaling pathway is located in a key regulatory position in connection with multiple functions of BAT.

The reduction in BAT function caused by obesity may be related to reduced VEGF expression. Adipose tissue is clearly vascularized, and its abundant blood vessels help transport FAs and secrete adipocytokines [32]. Obesity leads to adipose tissue becoming hypoxic, which causes imbalances in lipid metabolism, insulin resistance and local inflammatory responses [33], as well as capillary dysfunction and a decrease in the density of BAT [34]. As the only bona fide endothelial cell growth factor, VEGF (mainly VEGFa) regulates the proliferation and migration of endothelial cells, as well as vasodilation and vascular permeability [35, 36], and is therefore considered a potential treatment for adipose tissue damage and systemic metabolic diseases after obesity [37]. VEGFa is abundant in BAT [38], and decreased expression of VEGF caused by obesity may be related to functional hypoxia, leading to mitochondrial dysfunction and the accumulation of lipid droplet whitening of BAT [39].

Adrβ3 may play an important role in the regulation of BAT activity by VEGF. Obesity may reduce the number of capillaries in BAT and Adrβ3 expression, further increasing the mitochondrial production of reactive oxygen species (ROS) and autophagy [28]. Cold stimulates vascular endothelial cells to form capillaries, which upregulate VEGFa rapidly. Then, VEGFa binds to its receptor Flk-1 and activates downstream factors to increase the expression of COX2, a marker of mitochondrial heat production, further enhancing thermogenesis by BAT [40]. The changes in BAT induced by cold disappeared after resection of sympathetic nerves but could be restored by activators of noradrenaline (NE) and Adrβ3 [41].

Regular exercise leads to the accumulation of angiogenic signals that strengthen and increase the density of blood vessels in subcutaneous adipose tissue, but this effect is attenuated in obese organisms [42]. Acute exercise increases the level of VEGF mRNA in the WAT of rats on a HFD [32], and the angiogenic capacities of fat at different locations differ [43], but the effects of exercise on the blood vessels in BAT have rarely been reported. Activation of BAT by exercise is closely related to the activation of sympathetic nerves and secretion of NE. Our results showed that an agonist of Adrβ3 significantly reduced the lipid content in brown adipocytes and activated the VEGF-COX2 pathway, which was prevented by inhibition of COX2.

**Fig. 6:**
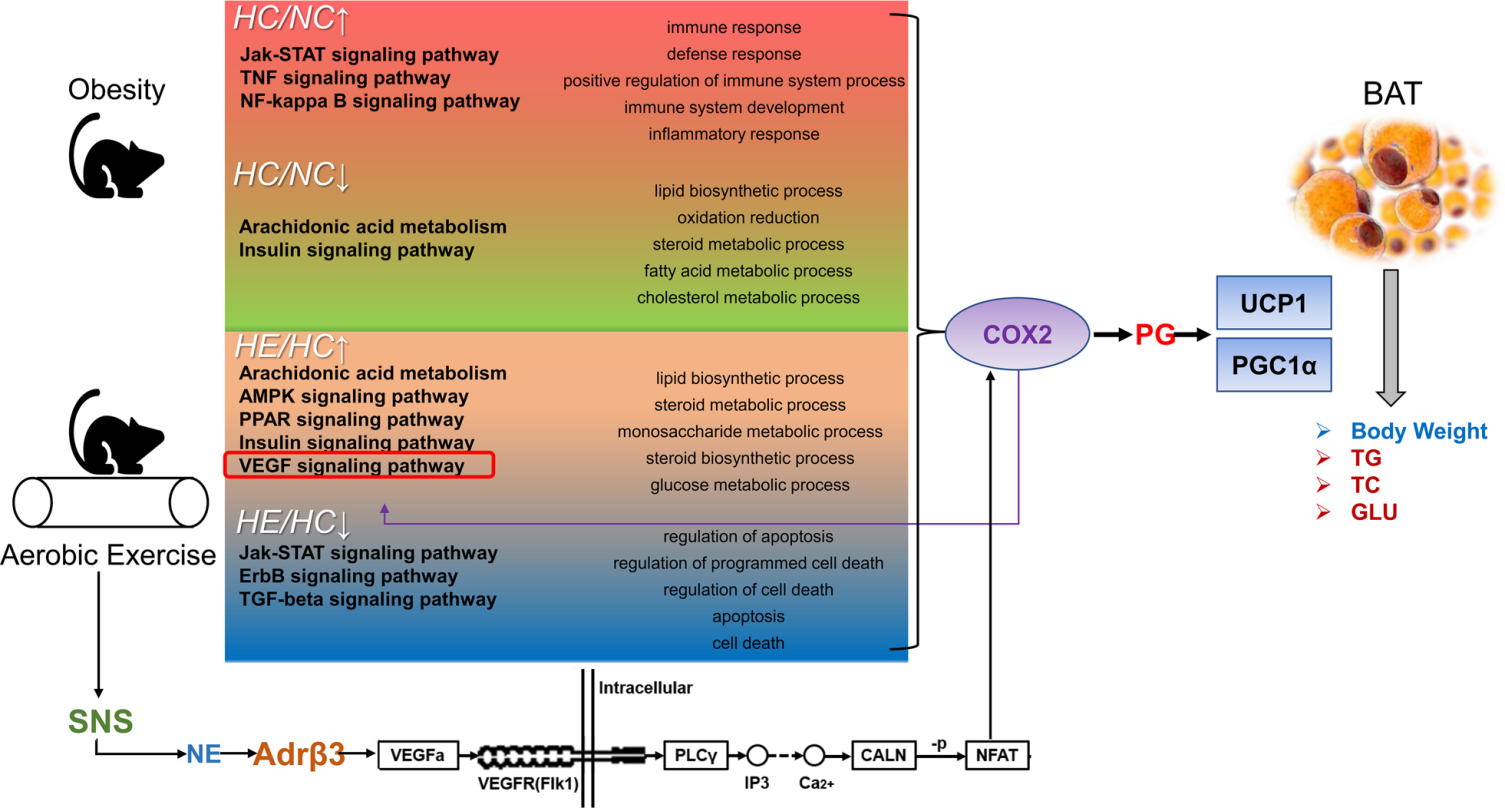
Changes in the BAT of obese individuals and regulation of BAT in these individuals by exercise

Activation of the VEGF pathway is dependent on the activation of Adrβ3 and its downstream COX2 [44], also known as prostaglandin synthetase, which can convert arachidonic acid into various prostaglandins (PGs), and increased levels of PG may be regarded as reflecting enhancement of the function of BAT [45, 46]. PGC-1α, another hallmark protein that reflects the activity of BAT, is the key regulator of mitochondrial biogenesis and oxidative metabolism [47]. Eight weeks of moderate treadmill exercise significantly reduced the expression of COX2 in the BAT of rats with HFD-induced obesity [48], increasing the resting metabolic rate and nonshivering thermogenesis (NST) and decreasing body fat [49]. After treatment with an Adrβ3 agonist, COX2 and PG levels were increased in WAT, and brown adipose tissue appeared. After treatment with analogs of PGE2 and PGI2, the mRNA levels of UCP1 and PGC-1α were increased in stromal-vascular fraction (SVF) cells derived from WAT, effects mediated by the PG receptor and PPARγ [50]. COX2 can be considered to be one of the effectors of Adrβ signaling in WAT and mediates the activation of inducible BAT (indBAT) [51]. The downstream PGI2/Ptgir/PPARγ/UCP1 signaling pathway could transform stromal progenitor cells into BAT, thereby contributing to energy consumption [52]. Figure 6 illustrates the entire regulatory process observed in our study.

Undoubtedly, the regulatory effect of exercise on BAT remains controversial. Numerous investigators have focused instead on the effects of exercise on WAT browning (caused by an increased number of brown or brite adipocytes), which may be more relevant, since the largest proportion of adipose tissue in our body by far is WAT. However, at present, the lack of uniform standards for the assessment of WAT browning and beige adipocytes makes studies in this area challenging, a situation that will improve as more appropriate experimental approaches are developed.

## Conclusion

HFD-induced obesity reduced the level of UCP1 and the mass of BAT, as well as the expression of genes related to glucose and lipid metabolism; increased the expression of genes related to inflammatory reactions; and caused the blood glucose and lipid levels to rise. Aerobic exercise increased BAT mass and the expression of genes related to glycolipid metabolism and anti-inflammatory processes in obese mice. Among the several pathways involved in this regulation process, COX2 in the VEGF pathway plays a key role.

## Data Availability

The datasets used and/or analyzed during the current study are available from the corresponding author on reasonable request.

## Abbreviations

HFD: High-fat diet
MS: Metabolic syndrome
BAT: Brown adipose tissue
WAT: White adipose tissue
UCP1: Uncoupling protein 1
FFA: Free fatty acid
SNS: Sympathetic nervous system
β-AR: β-Adrenergic receptor
TG: Triglyceride
TC: Total cholesterol
GLU: Glucose
Ptgs2/COX2: Prostaglandin-endoperoxide synthase 2/cyclooxygenase 2
Acsl5: Acyl-CoA synthetase long-chain family member 5
Fasn: Fatty acid synthase
Acaca: Acetyl-Coenzyme A carboxylase alpha
Fabp5: Fatty acid binding protein 5
Fbp1: Fructose bisphosphatase
Ptgis: Prostaglandin I2 (prostacyclin) synthase
VEGF: Vascular endothelial growth factor
Flk-1: Forms related to tyrosine kinase 1
PLCγ: Phospholipase C gamma
PGC-1α: Peroxisome proliferator-activated receptor α
